# Editorial: Unraveling Neuroprotective and Neurodegenerative Signals in Neurodegeneration

**DOI:** 10.3389/fnins.2016.00328

**Published:** 2016-07-13

**Authors:** Irving E. Vega, Timothy J. Collier

**Affiliations:** Department of Translational Science and Molecular Medicine, College of Human Medicine, Michigan State UniversityGrand Rapids, MI, USA

**Keywords:** neurodegeneration, neuroprotection, brain imaging, Alzheimer's disease, Parkinson's disease

Our understanding of the etiology of human neurodegenerative disorders is limited by the necessity to infer an active sequence of events, often expressed over protracted periods of time, from a collection of still-life images that often represent the end of the sequence with no clear view of how we got there. Adding to the collective confusion, it often is not clear whether our measures of nervous system structure and function at a given point in time represent active contributors to degeneration, the tombstones reflecting consequences of degeneration, active attempts to protect the nervous system, products of failed protection, or unrelated events. As a result, we rely on observations and manipulations of fragments of the process in experimental models and human samples, pieced together to create versions of what is possible. In this collection of articles, the iterative process is on full display, with a focus on Alzheimer's disease, Parkinson's disease, and related disorders, and reports ranging from tools to mechanisms to treatment.

## Alzheimer's disease and related disorders

For decades, the relative contributions and timing of accumulation of β-amyloid peptides (senile plaques) and oligomerization of the microtubule associated protein tau (neurofibrillary tangles) has been an open question in the etiology of Alzheimer's disease (AD). In this collection, Khan and Bloom review evidence suggesting that tau plays a central pathological role in AD as the mediator of β-amyloid neuronal toxicity. The authors discuss reports indicating that accumulation of β-amyloid oligomers affects tau's biological function, promoting microtubule instability, axonal transport defects, aberrant activation of kinases and synaptotoxicity. The authors argue that tau plays a central role in a “pathogenic signaling nexus that underlies AD.”

Sabbagh and Dickey discuss the known structural features and posttranslational modifications that contribute to tau function and toxicity in AD and other tauopathies. The authors review evidence indicating that posttranslational modifications and the function of chaperones influence tau structural dynamics, transitioning from its physiological conformation to pathological structures that lead to neurodegeneration.

The structural dynamics of tau is of great interest as a means to differentiate between normal and pathological tau. The contribution of Brelstaff et al. describes the use of pentameric formyl thiophene acetic acid (pFTAA), an amyloid specific fluorescent dye, to detect fibrillary tau in living neurons. The authors demonstrate that pFTAA can be used as a tool to assess the efficacy of compounds that block the accumulation of pathological tau and/or tau-mediated neurodegeneration in cultured neurons.

While, pFTAA specificity for amyloid proteins is of significant value in cell models, it will not distinguish between pathological β-amyloid and tau proteins in AD brain, limiting applications to *in vivo* imaging. Shimojo et al. review the advances in PET and fluorescence imaging as tools for the diagnosis of AD. The authors discuss the advantages of these techniques for detecting and dissecting disease progression in living organisms. The authors also discuss the limitations and pitfalls of PET and fluorescence imaging, providing a technical framework to improve the available technology.

Despite progress in the understanding of structural dynamics and detection of β-amyloid and tau in the context of the disease state, it remains unclear if the observed pathological structures are toxic or neuroprotective. Both pathological hallmarks also have been detected in cognitively normal individuals, suggesting that compensatory plasticity could mask the undergoing pathological process and, avert clinical presentation. Here, Avila et al. propose an alternate neuronal axis to explain how cognitive capacity is maintained in asymptomatic individuals with plaques and tangles. The authors argue that activation of the “default mode network (DMN)” bypasses the role of the AD-damaged entorhinal cortex, preserving function.

However, the progression of pathological processes could lead to synaptotoxicity, affecting the compensatory role of the DMN and contributing to clinical presentation. In this context, the role of hyperactivation of NMDA receptors and increased glutamate in AD is reviewed by Lewerenz and Maher. The authors extensively discuss evidence supporting the role of glutamate toxicity in neurodegeneration.

Study of other pathophysiologies associated with AD, in addition to β-amyloid and tau, hold potential to lead to development of tools that contribute to the understanding, diagnosis and treatment of the disease. For example, the contribution by Vega reviews published work that links the novel amyloid EFhd2 protein to AD and other neurodegenerative diseases. EFhd2 is a calcium binding protein which exhibits altered expression in AD and is found to co-aggregate with tau. Weinberg et al. present evidence that specific microRNAs may provide neuroprotection from β-amyloid toxicity through regulation of translation of specific RNA transcripts. In this study, they focus on sirtuin 1 and its neuroprotective role in β-amyloid toxicity. Thus, the understanding of alternate molecular mechanisms could contribute to unravel the pathophysiology of AD and related disorders.

## Parkinson's disease and related disorders

Like AD, Parkinson's disease (PD) is a multi-factorial syndrome that likely arrives at its clinical phenotype via a variety of combinations of risk factors and molecular pathways. One consistent feature of post-mortem PD brain is evidence of inflammation. Whether neuroinflammation contributes to PD neurodegeneration as cause, effect, or both, remains a topic of debate. Here, Walker et al. present findings from a quantitative multiplex protein analysis of 160 molecules, including those associated with inflammation, in substantia nigra (SN) and striatum of neuropathological confirmed cases of PD, incidental Lewy body disease (ILBD) and controls. The patterns of bidirectional changes in inflammation and growth factor proteins differ in SN and striatum, including novel changes in markers suggestive of reduced vascular integrity.

The accumulation of cytoplasmic protein aggregates containing alpha-synuclein (α-syn) in affected neurons is a neuropathological hallmark of PD. Delenclos et al. used a protein fragment complementation assay to monitor α-syn aggregates *in vivo*, providing a suitable model for therapeutic testing. However, whether α-syn aggregation elicits a toxic effect, neuroprotective mechanism, or both is debatable. While the association of α-syn with genetic forms of PD has been interpreted as disease-related toxic-gain-of-function, it is conceivable that sequestering the protein in aggregates results in toxic-loss-of-function. The contribution by Collier et al. presents new evidence from nonhuman primates consistent with the view that knock-down of endogenous α-syn in the nigrostriatal system faithfully reproduces the pattern of degeneration observed in PD.

Finally, the contribution of Lazzara and Kim reviews evidence addressing the neuroprotective potential of lithium for PD, AD and other neurodegenerative disorders. With the advent of drug repurposing as a strategy for a quicker path to clinical translation, many considered “dirty drugs,” due to side-effect profile and non-specificity for a single target, as the compounds best suited to treatment of complex, multi-factorial diseases. For the example of lithium, effects favoring reduced oxidative stress, reduced inflammation, inhibition of apoptosis, increased authophagy and stimulation of neurotrophic factors all address interacting systems implicated in neurodegenerative disease. Perhaps, for complex syndromes, drugs with highly focused targets may not be the best approach to treatment. The alternate approach of “dirty drugs” for complex diseases may hold significant value.

To make progress in our understanding of the complex etiology of AD, PD, and related disorders, it is clear that an appreciation of the web of interactions among multiple molecular pathways including neuroinflammation, energy metabolism, proteostasis and others will be critical to unraveling the mixed contributions of neuroprotective and neurodegenerative signals, supporting therapeutic efforts to harness one and limit the other.

## Author contributions

IV and TC reviewed the submissions and wrote the editorial.

### Conflict of interest statement

The authors declare that the research was conducted in the absence of any commercial or financial relationships that could be construed as a potential conflict of interest.

